# Port site recurrence, an unintended consequence of laparoscopic resection of ovarian cancer. A case report

**DOI:** 10.1016/j.ijscr.2019.07.024

**Published:** 2019-07-19

**Authors:** Paul H. Sugarbaker

**Affiliations:** Program in Peritoneal Surface Malignancies, MedStar Washington Hospital Center, Washington, DC, USA

**Keywords:** High grade serous cancer, Peritoneal metastases, Tumor cell entrapment, HIPEC, Intraperitoneal, Chemotherapy, Metastatic efficiency

## Abstract

**Background:**

Cancer surgery has as its goal complete clearance and absolute containment of the malignant disease as it is being resected. Any aspect of the procedure which allows the escapes of malignant cells may result in disease recurrence.

**Methods:**

The patient presented to an emergency room with left-sided abdominal pain. Pelvic sonogram suggested the diagnosis of a ruptured left ovarian cyst. A uterine biopsy showed high grade serous carcinoma. Definitive treatment of the primary ovarian cancer wasen bloc laparoscopic resection of her uterus, Fallopian tube and ovaries.

**Results:**

CT showed port site recurrence in both right and left upper abdominal trochar sites. The right-sided port site recurrence was through the fascia just lateral to the right rectus muscle and was small. A much larger port site metastases was present at the right trochar site that was through the left rectus muscle.

**Conclusion:**

The incidence of midline and lateral port site recurrence after laparoscopy for diagnosis or resection of ovarian cancer has not been determined. Limitation of trochar sites to the midline may reduce the extent of abdominal wall disease spread.

## Highlights

• The goal of cancer surgery is complete removal and absolute containment of the malignant process.
• Laparoscopy performed with cancer cells within the peritoneal space may cause cancer progression within trochar sites.
• This unintended adverse consequence of laparoscopy may be reduced by limiting trochar sites to the midline.
• Prospective data regarding the incidence of trochar site disease is not available but it does continue to occur.

## 1 Introduction

Every cancer surgeon must be aware that the surgical procedure used to resect primary cancer is part of the natural history of the disease process. Although cancer resection has as its goal complete curative resection of the malignancy, only a proportion of patients are cured. There may be situations where the surgical procedure with intention to completely resect and absolutely contain the disease results in dissemination of cancer cells. One surgical procedure reported to be oncologically flawed is morcellation of uterine leiomyosarcoma [1]. Unfortunately, if a uterine leiomyosarcoma, thought to be leiomyoma, is laparoscopically resected and then morcellated or sliced to facilitate extraction, sarcomatosis could result [2]. Recently, laparoscopic resection of early stage cervical cancer has been reported to result in a 65% higher risk of death as compared to open surgery. Within four years of open surgery 5.3% of women had died as compared to 9.1% of patients having a minimally invasive procedure [3]. Inadvertent spread of microscopic disease by some or many steps of the laparoscopic procedure could result in inadequate containment of cancer. Although studies show in randomized controlled trials that open and minimally invasive colon cancer resection have equivalent survival [4], port site metastases continue to be reported [5]. In this case report we follow the clinical course of a patient with early ovarian cancer who has a laparoscopic resection of her primary disease at an academic cancer center. Progression of disease in both right and left upper abdominal ports required another extensive reoperative procedure.

## 2 Materials and methods

Data on this patient was prospectively recorded and then retrospectively reviewed at an academic institution. This research work has been reported in line with the SCARE criteria [6]. This study was registered as a case report on the www.researchregistry.com website with UIN 4797.

## 3 Patient presentation

In June 2012, a 62 year old woman complained of left-sided abdominal pain. In an emergency room a diagnosis of ruptured ovarian cyst was made. Her gynecologist performed a uterine biopsy which showed high grade serous carcinoma. Stage of disease was International Federation of Gynecologic Oncology (FIGO) IIc with positive peritoneal cytology.

In August 2012, laparoscopic total hysterectomy with removal of adnexal structures, omentectomy, and para-aortic lymph node biopsy was performed. The upper abdomen was visually free of disease. Disease was confined to the pelvis. Postoperatively, modified FIGO stage was IC3 because of ovarian capsule rupture prior to surgery and positive peritoneal cytology. Postoperatively, she received intravenous paclitaxel and carboplatin for 6 cycles.

In February 2015, colonoscopy was performed because of rectal bleeding. Biopsy of a lesion on the right side of the colon showed serous carcinoma.

In April 2015, CT showed approximately 4 sites of disease progression. Cytoreductive surgery was performed for 11 h. It included peritoneal stripping of the undersurface of the right and left hemidiaphragms, completion greater omentectomy, small bowel resection, extended right colon resection, and pelvic peritonectomy. Intraoperatively, the patient received hyperthermic intraperitoneal chemotherapy (HIPEC) with cisplatin and doxorubicin plus systemic ifosfamide and 2-mercaptoethane sulfonate Na (MESNA). HIPEC was performed before closing the partial resection of the right hemidiaphragm, before the ileocolic and small bowel anastomoses and before abdominal closure [7]. The HIPEC was by the open technique [8]. A Tenckhoff catheter was placed for early postoperative intraperitoneal chemotherapy (EPIC) with paclitaxel [9]. Postoperatively, a deep venous thrombosis occurred after hospital discharge which was successfully treated with anticoagulation.

**Fig. 1:**
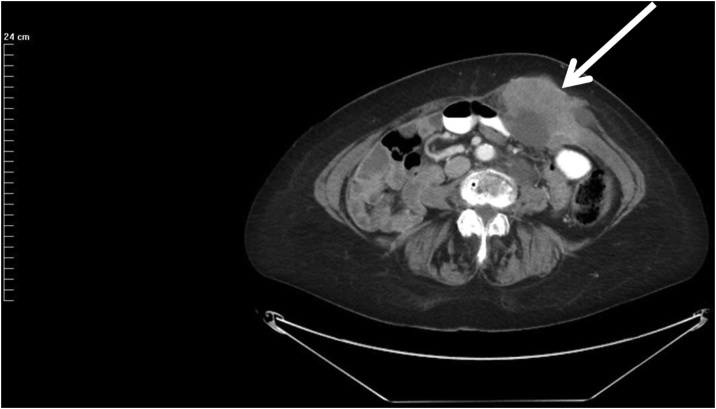
CT cut through the left mid-abdominal laparoscopy port site metastases. The entire thickness of the left rectus muscle appears to be infiltrated by serous ovarian cancer. The skin and subcutaneous tissue beneath the skin are not involved. The bowel directly beneath the mass does not appear to be invaded.

**Fig. 2:**
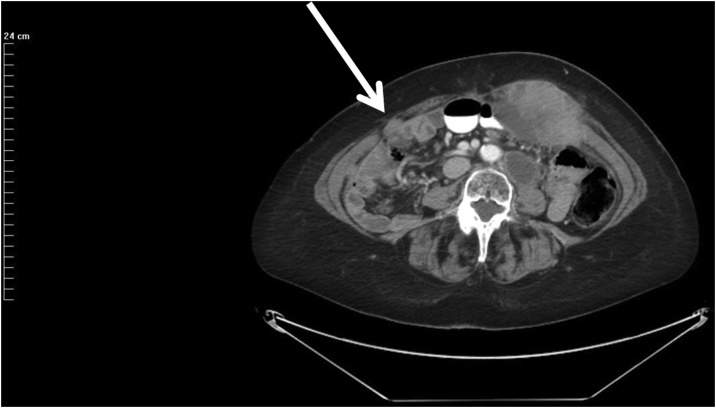
CT cut through the right mid-abdominal laparoscopy port site metastases. The anatomic location is through the fascia at the origin of the anterior and posterior rectus sheath. Although the left and right port site were tumor cell contaminated at the same time there is a marked size discrepancy. Progression of a left para-aortic lymph node is seen on this CT cut.

In January 2019, the CA-125 began to increase rapidly to 222 (upper limit of normal = 21). A CT showed a left-sided full thickness abdominal mass located directly beneath the left upper abdomen laparoscopy port site (Fig. 1). The mass measured 9 × 6.5 × 10 cm. The skin over the mass was not involved by CT. A second abdominal wall mass 2 × 1 cm was present immediately beneath the right upper abdominal laparoscopy port site through fascia at the lateral border of the right rectus muscle (Fig. 2).

In April 2019, the masses within the left rectus muscle progressed and required hospitalization for control of pain. An expanding left para-aortic mass was also present. Plans to resect the trochar site disease were delayed in order to begin additional systemic treatments.

## 4 Discussion

There is no doubt that laparoscopic staging of ovarian cancer is being more frequently used [10]. Also in selected patients, as in this case report, the definitive debulking procedure is performed by a minimally invasive procedure [11]. The incidence of port site disease subsequent to laparoscopic resection has never been prospectively reported. The progression of abdominal wall disease at port sites is clinically overshadowed, in nearly all patients, by progression of cancer within the abdomen and pelvis. Only as the patient reported here continued to remain disease-free after cytoreductive surgery and perioperative intraperitoneal chemotherapy was the port site disease thought relevant for a surgical intervention. It is completely possible that more effective the control of peritoneal metastases causes port site recurrence to be more frequently observed and require excision.

In this case report we have characterized port site metastases as an unintended consequence of laparoscopy to resect ovarian cancer. However, port site metastases are not restricted to ovarian cancer minimally invasive surgery. With uterine leiomyosarcoma, laparoscopic resection and then morcellation or slicing resulted in sarcomatosis in a majority of patients [1]. These cancers were inadvertently disseminated thinking that a benign leiomyoma was being morcellated when the mass was actually sarcoma. With early stage cervical cancer, laparoscopic resection resulted in 9.15% of patients dead of disease at 4 years as compared to 5.3% of patients having laparotomy [3]. A prospective study to determine the incidence of port site metastases in patients following laparoscopic resection has not been reported. Wexner and Cohen reviewed the early experience with laparoscopic resection of colon cancer [12]. Early on a lack of precise technology with colon cancer resection resulted in an unacceptable high incidence of port site metastases. As these patients were studied it became evident that port site cancer nodules, almost always, were an indication of widespread peritoneal metastases and guarded prognosis [5]. After refinements in laparoscopic techniques, Nelson et al. reported in a randomized controlled trial that survival the laparoscopic colon resection compared to survival after open colon resection [4]. However, Kwong and colleagues showed that port site metastases do continue to occur after laparoscopic or robotic resection outside of a clinical trials setting. Another disease where port site metastases has been repeatedly reported is peritoneal mesothelioma [13]. The diagnosis of peritoneal mesothelioma is seldom made from a paracentesis so laparoscopy is usually employed to make a diagnosis [14]. An unknown proportion of these patients will develop port site disease.

The location of the laparoscopic port site may be important in the progression that is observed. In our patient there was a 9 cm diameter mass in the left-sided port that seemed to pass through the middle of the left rectus muscle. On the right side the disease progression was lateral to the right rectus muscle and through the aponeuorosis. Yan and Sugarbaker observed that port site recurrence in the rectus muscle was likely to spread along muscle fascicles. Definitive resection required complete or near complete removal of that rectus muscle [15]. That occurred in our patient. However, the small and more contained port site recurrence on the right was through fascia at the lateral aspect of anterior and posterior rectus sheath. Diagnostic laparoscopy in patients who may have peritoneal metastases should be through the linea alba to minimize the extent of port site disease progression. Also, if midline ports are used exclusively, the trochar site can be excised a part of a midline abdominal incision.

The mechanism whereby port site metastases occur has never been clearly explained. Free cancer cells within the peritoneal space are necessary. A tunnel through the abdominal wall that allows access to abdominal wall tissue surfaces is necessary. Small amounts of peritoneal fluid containing cancer cells may be forced along the trochar site by intraabdominal pressure from CO_2_ insufflation. Slight in and out or side to side movement of the trochar sheath may help deposit cancer cells on the lower aspect of the trochar tunnel. Postoperatively, ascites fluid may drain from the peritoneal cavity out through the port site and thereby seed the traumatized tissues. It has been well established that traumatized tissues are a site for high metastatic efficiency [16]. Sugarbaker et al. noted that radiologically, port site recurrence by CT has a triangular configuration with the base at the level of the peritoneum [17]. The largest volume of disease was at the entrance to the port site tunnel. After completion of laparoscopy peritoneal fluid contaminated by cancer cells may enter the peritoneal aspect of the port site and infiltrates these tissues with the largest number of cancer cells. Skin and subcutaneous tissue are less frequently invaded as was seen in the patient presented.

In conclusion, laparoscopic resection of an ovarian cancer resulted in disease implantation within the abdominal wall. Extensive abdominal and pelvic treatments which involves cytoreductive surgery and perioperative chemotherapy adequately treated the intraabdominal component of the disease so that isolated port site metastases required resection. Surgical technology to prevent port site metastases have not been as yet described. However, limiting the ports to the midline may limit the disease progression within the abdominal wall and allow a less extensive surgical procedure if resection is required.

## Funding

Data management and secretarial support provided by Foundation for Applied Research in Gastrointestinal Oncology.

## Ethical approval

Local IRB-approval for this case report was not required:

MedStar Health Institutional Review Board has determined that a case report of less than three [3] patients **does not meet the DHHS definition of research** (45 CFR 46.102(d)(pre-2018)/45 CFR 46.102(l)(1/19/2017)) **or the FDA definition of clinical investigation** (21 CFR 46.102(c)) and therefore are not subject to IRB review requirements and **do not require IRB approval**.

This case report is of 1 patient.

## Consent

Written and signed consent was obtained from the patient.

## Author contribution

Paul H. Sugarbaker, MD: study concept or design, data collection, data analysis or interpretation, writing the paper.

## Registration of research studies

This study was registered as a case report on the www.researchregistry.com website with UIN 4797.

## Guarantor

Paul H. Sugarbaker, MD.

## Provenance and peer review

Not commissioned, externally peer-reviewed.

## Declaration of Competing Interest

Paul H. Sugarbaker has no conflicts of interest to declare.
